# High Sensitivity In Vivo Imaging of Cancer Metastasis Using a Near-Infrared Luciferin Analogue seMpai

**DOI:** 10.3390/ijms21217896

**Published:** 2020-10-24

**Authors:** Jun Nakayama, Ryohei Saito, Yusuke Hayashi, Nobuo Kitada, Shota Tamaki, Yuxuan Han, Kentaro Semba, Shojiro A. Maki

**Affiliations:** 1 Department of Life Science and Medical Bioscience, School of Advanced Science and Engineering, Waseda University, Tokyo 162-8480, Japan; h-yusuke@moegi.waseda.jp (Y.H.); y.han2@kurenai.waseda.jp (Y.H.); ksemba@waseda.jp (K.S.); 2 Division of Cellular Signaling, National Cancer Center Research Institute, Tokyo 104-0045, Japan; 3 School of Pharmacy, Tokyo University of Pharmacy and Life Sciences, Tokyo 192-0392, Japan; rsaito@toyaku.ac.jp; 4 Department of Engineering Science, Graduate School of Informatics and Engineering, The University of Electro-Communications, Tokyo 182-8585, Japan; kitada@uec.ac.jp (N.K.); stamaki95@yahoo.co.jp (S.T.); s-maki@uec.ac.jp (S.A.M.); 5 Center for Neuroscience and Biomedical Engineering, The University of Electro-Communications, Tokyo 182-8585, Japan; 6 Department of Cell Factory, Translational Research Center, Fukushima Medical University, Fukushima 960-1295, Japan

**Keywords:** in vivo imaging, near-infrared bioluminescence, luciferin analogue, metastasis

## Abstract

Bioluminescence imaging (BLI) is useful to monitor cell movement and gene expression in live animals. However, D-luciferin has a short wavelength (560 nm) which is absorbed by tissues and the use of near-infrared (NIR) luciferin analogues enable high sensitivity in vivo BLI. The AkaLumine-AkaLuc BLI system (Aka-BLI) can detect resolution at the single-cell level; however, it has a clear hepatic background signal. Here, to enable the highly sensitive detection of bioluminescence from the surrounding liver tissues, we focused on seMpai (C_15_H_16_N_3_O_2_S) which has been synthesized as a luciferin analogue and has high luminescent abilities as same as AkaLumine. We demonstrated that seMpai BLI could detect micro-signals near the liver without any background signal. The solution of seMpai was neutral; therefore, seMpai imaging did not cause any adverse effect in mice. seMpai enabled a highly sensitive in vivo BLI as compared to previous techniques. Our findings suggest that the development of a novel mutated luciferase against seMpai may enable a highly sensitive BLI at the single-cell level without any background signal. Novel seMpai BLI system can be used for in vivo imaging in the fields of life sciences and medicine.

## 1. Introduction

Bioluminescence imaging (BLI) is used to monitor the behavior of cells and molecules in animal studies [1,2]. In cancer research, the in vivo BLI system with luciferase is used for the quantification of tumor volume, monitoring gene expression and detection of cancer metastasis [3,4,5,6,7,8]. However, luminescence of visible light (450–600 nm) including D-luciferin’s wavelength (560 nm) is absorbed by tissues. In particular, high sensitivity in vivo BLI in deep tissues is difficult by absorption of short wavelength bioluminescence. Recent studies showed that luciferase-luciferin analogue reactions at near-infrared (NIR) wavelengths enable a highly sensitive in vivo BLI [9,10]. Furthermore, the AkaLumine-AkaLuc bioluminescence imaging system (Aka-BLI) at NIR wavelengths can detect resolution at the single-cell level in several live animals [11]. NIR imaging techniques using luciferin analogues are used in oncology, neuroscience and ethology [2,12,13,14].

However, AkaLumine and AkaLumine-HCl (TokeOni) have a clear hepatic background signal, and it has a problem in monitoring weak luminescence signals from micro-metastases nearby the liver [15]. This background signal only occurs with the administration of AkaLumine or TokeOni. This suggests that luciferin analogues emit micro-luminescence by undergoing metabolism in the liver. To monitor micro-signals from those tissues, the background signal should be reduced to ensure a highly sensitive BLI. In general, hydrophobic molecules tend to accumulate in the liver [16], we hypothesized that hepatic accumulation is caused by the hydrophobicity of TokeOni.

In this study, to improve the background signal in NIR BLI, we focused on a luciferin analogue seMpai (C_15_H_16_N_3_O_2_S), which demonstrates a high level of luminescence similar to that of AkaLumine and TokeOni [17]. Since seMpai has increased hydrophilicity in solution compared to TokeOni, it has the potential to decrease accumulation in the liver. We performed the comparative analysis of luciferin analogues in cancer metastasis model using in vivo imaging system (IVIS). We propose that seMpai BLI can suppress the background signals in the liver, enabling the detection of micro-metastases in live animal models.

## 2. Results and Discussions

**Figure 1:**
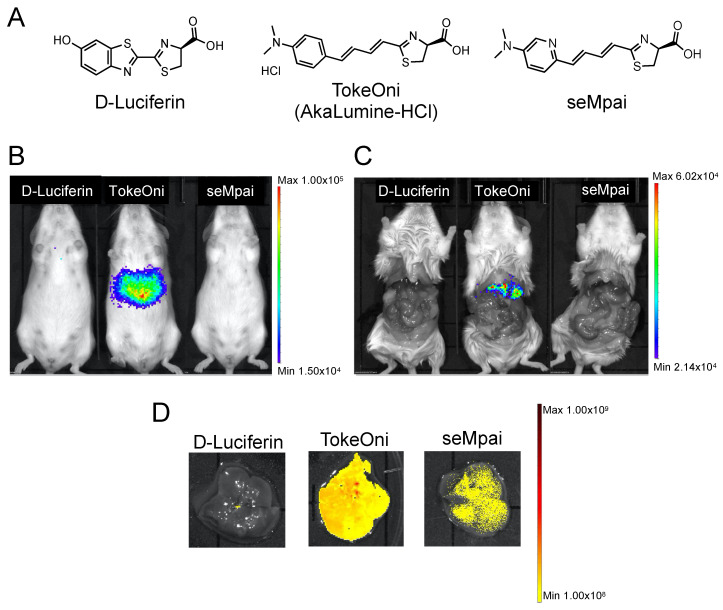
Hepatic background signals of luciferin analogues. Structural formulae of firefly D-luciferin and luciferin analogues (**A**). Bioluminescence imaging (BLI) of D-luciferin and luciferin analogues using non-transplanted female mice (**B**). BLI in mice with an open peritoneum (**C**). Ex vivo fluorescence imaging of the harvested livers using a 670 nm emission filter and an open excitation light (**D**). The scale bar’s unit is the radiance (p/s/cm^2^/sr).

First, we compared the background signals of the luciferin analogues with that of firefly D-luciferin. D-luciferin, TokeOni, and seMpai were administered to female non-transplanted Rag2-/- mice (Figure 1A). Hepatic background signals were detected only in TokeOni-administered mice (Figure 1B,C). This result suggested that seMpai can detect micro-signals without any hepatic background signal. Next, we performed ex vivo fluorescence imaging of harvested livers from the administered mice using a 670 nm emission filter. The emission maximum of seMpai was at 675 nm, and its in vivo bioluminescence activity was similar to that of AkaLumine and TokeOni [17]. Interestingly, TokeOni and seMpai were detected in the harvested liver; however, the amount of accumulated seMpai was low as compared to that of TokeOni (Figure 1D). Our results suggest that the decreased accumulation of seMpai in the liver reduced the background micro-signals. Thus, seMpai could perform a highly sensitive imaging of liver micro-metastases without any background signal. According to chemical properties, hydrophobic molecules have a high ability to accumulate in the liver [16]. Therefore, it is considered that the increased hydrophilicity of seMpai decreased its translocation to the liver.

**Figure 2:**
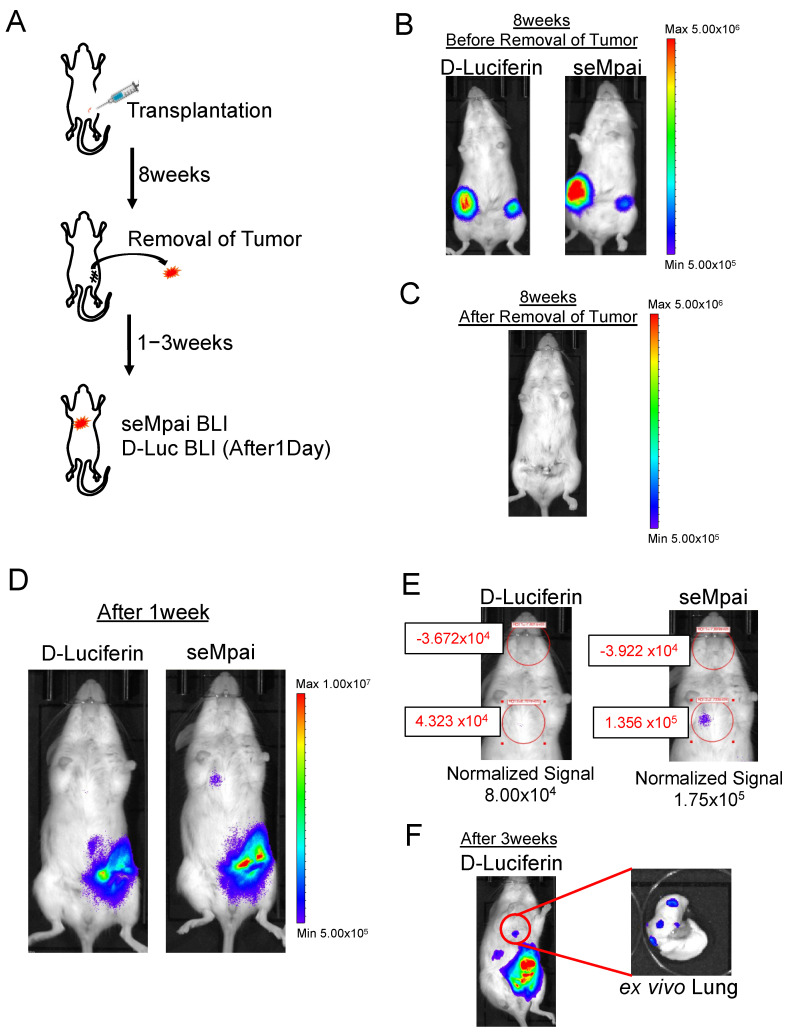
Detection of micro-metastases in the orthotopic transplantation model. Scheme of orthotopic transplantation using NMuMG-ERBB2-metastatic cells (**A**). Eight weeks after transplantation, comparative analysis of D-luciferin BLI and seMpai BLI. Before removal of the primary tumor (**B**) and after removal of the primary tumor (**C**). One week after the removal, comparative analysis of D-luciferin BLI and seMpai BLI. The same mouse was used to perform BLI of D-luciferin and seMpai (**D**). ROI measurement of micro-signal from the pleural lesion in D-luciferin BLI and seMpai BLI (**E**). seMpai could detect micro-metastases in the breast cancer metastasis model. Three weeks after removal, D-luciferin BLI could detect the micro-signal from the lung metastases (**F**). The scale bar’s unit is the radiance (p/s/cm^2^/sr).

Next, we performed orthotopic injection using NMuMG-ERBB2-metastatic cells (Figure 2A). When we monitored D-luciferin BLI and seMpai BLI 8 weeks after the transplantation, metastases could not be detected (Figure 2B,C). One week after the removal of a primary tumor, seMpai BLI could detect pulmonary micro-metastases, whereas D-luciferin BLI could not detect pulmonary micro-metastases (Figure 2D). The detected signal by seMpai BLI was in the 10^5^ order, suggesting that it is difficult to detect the micro-signal from metastases by the hepatic background of TokeOni (Figure 2E). Three weeks after the removal of the primary tumor, D-luciferin BLI could detect the signal from the same sites. This micro-signal originated from lung metastases in the transplanted mouse (Figure 2F). At the fourth fat pad, bioluminescence was detected from residual small primary tumors and invasive cancer cells. NIR BLI effectively performs deep tissue imaging [17], but a strong signal was also detected in shallow mammary gland tissue compared with D-luciferin imaging. Our results show that seMpai enabled the detection of micro-metastases without any liver-specific background signal in a breast cancer metastasis model.

**Figure 3:**
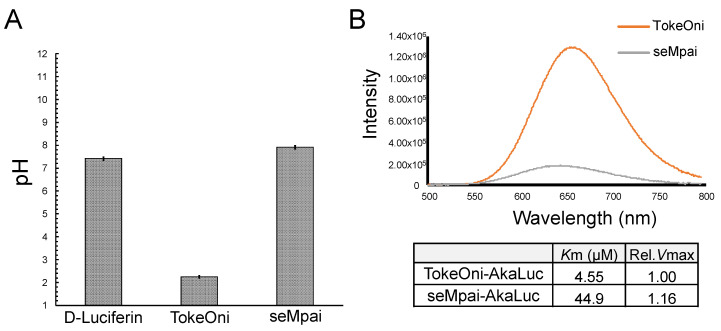
Measurements of pH value and bioluminescence of luciferin analogues with AkaLuc. pH values of 10 mM solutions of D-luciferin, TokeOni and seMpai (*n* = 3) (**A**). In vitro reaction of luciferin analogues with AkaLuc (**B**). The table shows the value of Michaelis constant (*K*_m_) and relative maximal velocity (rel. *V*_max_) for luciferin analogues with AkaLuc.

After the intraperitoneal administration of TokeOni in mice, a rapid fall in heart rate was observed. Therefore, we investigated the pH values of the administered solutions (Figure 3A). The pH of D-luciferin, TokeOni, and seMpai was 7.42, 2.25, and 7.91, respectively. These results suggest that high concentration (over 10 mM) of TokeOni caused cardiac damage by inducing acidosis. On the other hands, D-luciferin and seMpai did not induce acidosis owing to their neutral pH at high concentrations. The solution of seMpai was slightly unstable; therefore, its imaging sensitivity may be further improved by optimizing its protocol.

AkaLumine and TokeOni can realize single-cell resolution BLI by using AkaLuc which is mutated luciferase adjusted for AkaLumine [11]. Since seMpai and TokeOni are structurally similar, we measured the in vitro luminescence of seMpai-AkaLuc. seMpai-AkaLuc BLI demonstrated a low intensity luminescence and short wavelength as compared to those of TokeOni-AkaLuc (Figure 3B). The K_m_ of seMpai was 10 times higher than that of TokeOni, indicating that seMpai had a low affinity to AkaLuc as compared to that of TokeOni. Interestingly, seMpai substituted a nitrogen atom in the TokeOni moiety, modifying the enzyme-substrate reaction of AkaLuc. On the other hand, the V_max_ of seMpai was high, similar to that of TokeOni. This suggests that high concentrations of seMpai can strongly react with AkaLuc. However, it is impossible to increase the concentrations of luciferin analogues for in vivo imaging. Therefore, the development of a novel luciferase for seMpai will be helpful for more high sensitivity BLI.

## 3. Materials and Methods

### 3.1. Synthesis of seMpai

seMpai was synthesized by seven steps as previously reported [17], which was prepared from commercially available aminonitrile as starting substrate. Finally, the crude products were purified by automated flash chromatography (Smart Flash EPCLC AI-580S, ULTRAPACK COLUMNS C18, Yamazen Corp., Osaka, Japan,) to yield seMpai.

### 3.2. Cell Culture

Normal murine mammary gland (NMuMG) epithelial cell line (kindly provided by Dr. Miyazawa, University of Yamanashi, Yamanashi, Japan) were cultured in Dulbecco’s modified Eagle’s medium (DMEM; FUJIFILM Wako, Osaka, Japan) supplemented with 10% heat-inactivated fetal bovine serum (FBS, Nichirei Biosciences Inc., Tokyo, Japan), 100 U/mL penicillin (Meiji-Seika Pharma Co., Ltd., Tokyo, Japan), 100 µg/mL streptomycin (Meiji-Seika Pharma Co., Ltd.), 10 µg/mL insulin (FUJIFILM Wako) and 4.5% glucose at 37 °C with 5% CO_2_. NMuMG-ERBB2V659E-luc cell expressing a cancer-related gene located on *ERBB2* amplicon 17q12.21 (named as NMuMG-ERBB2-metastatic cell) was produced as previously described [8,18].

### 3.3. Animal Studies

Female *Rag2-/-* mice (12 weeks old) were used to detect the background signals produced by luciferin analogues. In this process, 30 mM TokeOni (AkaLumine-HCl; kindly provided by Kurogane Kasei Co., Ltd., Aichi, Japan) and 60 mM seMpai were administered intraperitoneally into the mice and the BLI was performed using in vivo imaging system (IVIS Lumina XRMS; Perkin-Elmer, Waltham, MA, USA). Additionally, 200 µL firefly D-luciferin (15 mg/mL, 47.1 mM; Gold Biotechnology, Inc., St. Louis, MO, USA) was administered intraperitoneally into the mice.

Female *Rag2-/-* mice (6–8 weeks old) were used for cancer metastasis model with in vivo BLI. 1.0 × 10^6^ cells/10 µL PBS (-) (FUJIFILM Wako) were transplanted into the fourth mammary fat pad as orthotopically injection. When tumor volume reached 300 mm^3^, primary tumors at fat pad were removed as previously described [5]. Comparative analysis of D-luciferin and seMpai was performed with the same amount of substrate (6 µmol/body) and the same transplanted mouse. Conditions of in vivo and ex vivo BLI were 30 s exposure time, binning of 2 and F-stop of 1. Ex vivo fluorescence imaging of harvested livers was performed using an open excitation light and 670 nm emission filter. The quantification of BLI signal was performed by the ROI measurement tool of Living Image software (Perkin-Elmer). All animal experiments were approved by the Animal Committee of Waseda University. Genetic and animal experiments were approved by the Ethics Committee of Waseda University (WD19-058, 2019-A068 in 2019 April).

### 3.4. pH Measurement of Luciferin Analogues

The pH values at 25 °C of D-luciferin potassium salt (Promega, WI, USA), seMpai in PBS (pH 7.4) and TokeOni in ultrapure water were determined by the pH meter (HORIBA, Kyoto, Japan). The luciferin analogues were dissolved in 10 mM for pH measurement.

### 3.5. Bioluminescence Measurement of seMpai-AkaLuc Reaction

seMpai was reacted with the mutant luciferase AkaLuc (kindly provided by Dr. Iwano, RIKEN, Saitama, Japan), and bioluminescence of the reaction mixture was measured. seMpai was dissolved in 50 mM potassium phosphate buffer (KPB, pH 6.0), AkaLuc was dissolved in 50 mM KPB (pH 8.0) containing 35% glycerol, and Mg-ATP was dissolved in ultrapure water. A bioluminescence reaction was initiated by injecting 10 μL of 10 mM Mg-ATP into a mixture containing 5 μL of 100 μM substrate solution, 5 μL of 0.1 mg/mL luciferase solution, and 5 μL of 500 mM KPB (pH 8.0). Emission spectra were measured on the AB-1850 spectrophotometer (ATTO, Tokyo, Japan) in the range of 400–790 nm (slit width: 1.0 mm, exposure time: 3 min). Light intensity was determined as the intensity of emission spectrum at the maximum wavelength. For Lineweaver–Burk analysis, light emission from the bioluminescence reaction was monitored with the AB-2270 luminometer (ATTO) for 30 s with sampling intervals of 0.1 s. Emission intensities were expressed as counts per second. The bioluminescence reaction was initiated by injecting 40 μL of 200 μM Mg-ATP into a mixture containing 20 μL of 0.1–500 μM substrate solution, 20 μL of 0.01 mg/mL luciferase solution, and 20 μL of 500 mM KPB (pH 8.0) at 25 °C. Final concentrations of the substrates varied from 0.02 to 100 μM. Values of Michaelis constant (*K*_m_) and maximal velocity (*V*_max_) of the substrates were determined from the integrated emission intensity values, and they were calculated using Lineweaver–Burk plots and the Enzyme Kinetics Module commercially available SigmaPlot 13.0 software package (Systat Software Inc., CA, USA), as previously described [17].

## 4. Conclusions

Our study suggests that seMpai can detect micro-metastases without any background signal. In addition, development of a novel mutated luciferase against seMpai may enable a highly sensitive BLI at the single-cell level without any background signal. The future research should focus on establishing a universal BLI protocol for seMpai and developing an optimized luciferase for seMpai, similar to that of the AkaBLI system. Establishment of seMpai BLI technology may be used for in vivo analyses in the fields of life sciences and medicine.

## Abbreviations

BLI	Bioluminescence imaging
NIR	Near-infrared
Aka-BLI	AkaLumine-AkaLuc bioluminescence imaging
HCl	Hydrochloric acid
ERBB2	Erb-b2 receptor tyrosine kinase 2
PBS	Phosphate Buffered Saline
KPB	Potassium phosphate buffer
ATP	Adenosine triphosphate
ROI	Region of interests
